# Isolation, Sphalerite Bioleaching, and Whole Genome Sequencing of *Acidithiobacillus ferriphilus* QBS3 from Zinc-Rich Sulfide Mine Drainage

**DOI:** 10.3390/life15050792

**Published:** 2025-05-15

**Authors:** Kan Wang, Yuandong Liu, Run Liu, Wissal Belqadi, Weimin Zeng, Runlan Yu, Xueling Wu

**Affiliations:** 1 School of Minerals Processing and Bioengineering, Central South University, Changsha 410083, China; 225611016@csu.edu.cn (K.W.); abcliurun@sina.com (R.L.); wissal.belqadi@edu.uiz.ac.ma (W.B.); zengweimin1024@126.com (W.Z.); yrl206023@csu.edu.cn (R.Y.); wxlcsu@csu.edu.cn (X.W.); 2 Key Laboratory of Biohydrometallurgy of Ministry of Education, Changsha 410083, China

**Keywords:** *Acidithiobacillus ferriphilus*, isolation, bioleaching, sphalerite, ferrous oxidation, sulfur oxidation

## Abstract

The genus *Acidithiobacillus* has been widely used in bioleaching, and novel strains in this genus, such as *A. ferriphilus*, have also been confirmed to possess bioleaching capabilities. In this study, an *Acidithiobacillus ferriphilus* strain, QBS3, was isolated from zinc-rich sulfide mine drainage using the gradient dilution method. QBS3 is a Gram-negative, 1.3 µm rod-shaped bacterium with small red colonies. It showed a high iron oxidation efficiency of 0.361 g/(L·h) and a sulfur oxidation efficiency of 0.206 g/(L·d). QBS3 has sphalerite bioleaching ability; using QBS3 for pure sphalerite bioleaching, 18.8% of zinc was extracted in 14 days at 1% pulp density. Whole genome sequencing was performed on QBS3. Functional prediction showed that 9.13% of the genes were involved in replication, recombination, and repair. Bioleaching-related genes were analyzed, including iron and sulfur oxidation genes, and carbon and nitrogen fixation genes. For iron oxidation, the Cyc2→RusA pathway and Iro→RusB pathway were found in QBS3. In terms of sulfur oxidation, QBS3 has an incomplete SOX system and lacks the SDO gene, but Rho and Trx may complement the SOX system, enabling QBS3 to oxidize sulfur. QBS3 has multiple sets of carbon fixation genes, and nitrogen fixation genes were also identified. A hypothetical sphalerite bioleaching model is proposed; this study provides a theoretical basis for the zinc sulfide ore bioleaching industry.

## 1. Introduction

Bioleaching is a technique that uses microorganisms to extract metals from ores [1]. This technique is commonly used for sulfide mineral ores. One reason for this is that traditional pyrometallurgical methods for extracting metals from sulfide minerals are expensive and produce sulfur dioxide emissions, which may cause environmental pollution [2]. Another reason is that sulfide ores inherently contain sulfur and often iron, both of which can be metabolically harnessed as energy sources by most bioleaching microorganisms. [3]. In the bioleaching process, iron and sulfur in ores are oxidized by bioleaching microorganisms, generating ferric ions and acid. Iron is released in this step, and then target metals such as copper, zinc, and lead are released indirectly. Inert metals such as gold and silver cannot be oxidized in this way, but can be exposed and extracted using cyanide agents [4].

The effectiveness of bioleaching is mainly determined by bioleaching microorganisms. Bioleaching efficiency can be improved by screening better bioleaching strains and studying their mechanisms. Among all bioleaching microorganisms, iron- and sulfur-oxidizing bacteria are the most widely studied, due to their important roles in acid mine drainage and mineral oxidation [3]. The genus *Acidithiobacillus* is the most widely studied and widely used group of iron- and sulfur-oxidizing bacteria in bioleaching. These extremophiles are acid-tolerant, thrive in acidic environments (pH 1–2), and possess both iron and sulfur oxidation capabilities. Chalcopyrite bioleaching with *A. ferrooxidans* has already been implemented in industrial production in many countries and has achieved good results [5]. The iron and sulfur oxidation mechanisms of *A. ferrooxidans* were studied in depth after its strain ATCC 23270 [6] was sequenced and annotated. Related genes were mapped, and the functional proteins were expressed in vitro. These studies have clarified the biological oxidation mechanisms of iron and sulfur and significantly promoted the industrial application of *A. ferrooxidans* ATCC 23270.

Since then, many novel species have been discovered in the *Acidithiobacillus* genus. Three species with core characteristics similar to *A. ferrooxidans* are *A. ferrivorans*, *A. ferridurans*, and *A. ferriphilus* [7]. Among those species, *A. ferriphilus* is the most recently discovered and the least studied. While these species share general iron and sulfur oxidation mechanisms with *A. ferrooxidans*, they also show unique differences. Microbiological studies on these species are still incomplete and need improvement.

In this study, a high iron- and sulfur-oxidizing bioleaching bacterium, *Acidithiobacillus ferriphilus* QBS3, was isolated from zinc-rich sulfide mine drainage. Pure sphalerite without iron content was used for bioleaching with QBS3, and 8 g/L of ferrous ions was added to simulate sphalerite with iron content in order to observe the differences. Furthermore, the whole genome of QBS3 was sequenced, revealing its genomic information and identifying a large number of key genes related to bioleaching functions. Based on both the bioleaching experiments and genomic analysis, a theoretical model of sphalerite bioleaching was developed.

This study provides a theoretical basis for future research on zinc-bearing mineral bioleaching and shows the potential for industrial applications in zinc extraction using *A. ferriphilus* QBS3.

## 2. Materials and Methods

### 2.1. Site Description, Sampling, and Isolation of Bioleaching Bacteria

Strain QBS3 was isolated from an acid mine drainage sample collected from a zinc–lead-rich sulfide mineral waste mine site in Qibaoshan Mine, located at Liuyang City, Hunan Province (113°53′ E, 28°17′ N). The drainage sample was filtered through a high-pressure filtration system using a 0.22 μm organic microporous membrane to collect bacteria. Then, the membrane was sheared and washed with 9K medium (consisting of 3 g/L (NH_4_)_2_SO_4_, 0.5 g/L KH_2_PO_4_, 0.1 g/L KCl, 0.5 g/L MgSO_4_·7H_2_O, and 0.01 g/L Ca(NO_3_)_2_·2H_2_O) to enrich the bacterial population [8]. An amount of 1 mL of enriched bacteria sample and approximately 1 g of zinc–rich ore were added to 100 mL of 9K medium in a 250 mL conical flask. Additionally, 44.7 g/L FeSO_4_·7H_2_O was added as an energy source (final Fe^2+^ concentration was 9 g/L). After 60 days of incubation at 30 °C and 170 rpm in a shaking incubator (ZQZY-108B, Shanghai Zhichu Instrument, Co., Ltd. (Shanghai, China)), the growing strain was isolated through four generations of serial dilution culture. The bacterial culture broth was diluted serially in 10-fold increments to a final dilution factor of 10^9^. Then, 1 mL of different multiple diluents was inoculated into 9K medium with 44.7 g/L FeSO_4_·7H_2_O added. After 15 days of incubation, the culture medium with bacterial growth and the highest dilution factor was used for the next generation of serial dilution culture. After four generations, the culture was purified to obtain a pure culture and stored in cryovials containing 20% (*w*/*v*) PEG-2000 at −80 °C for future use. All reagents mentioned above were purchased from Macklin Biochemical Technology, Co., Ltd. (Shanghai, China).

### 2.2. Identification of the Bioleaching Strain

QBS3 was inoculated into 9K medium and cultured for 3 days. The culture was first centrifuged at 2000 rpm for 2 min to remove the sediment (mainly jarosite). The supernatant was then collected and centrifuged at 9000 rpm for 8 min to collect organisms. Genomic DNA was extracted using the TIANGEN Bacteria DNA Kit (Tiangen Biotech, Co., Ltd. (Beijing, China)), following the manufacturer’s instructions. Universal bacterial primers 27 F (5′-AGAGTTTGATCCTGGCTCAG-3′) and 1492 R (5′-GGTTACCTTGTTACGACTT-3′) were used for the polymerase chain reaction (PCR) of the 16S rRNA gene of QBS3. The amplified products were sent to Tsingke Biotechnology, Co., Ltd. (Changsha, China) for quality control and sequencing. The returned partial sequences were aligned using BLAST+ v2.14.1 on the NCBI GenBank (https://ncbi.nlm.nih.gov/, accessed on 15 November 2023) to identify the species with the highest homology. Then, the 16S rRNA gene sequences of the top seven most homologous different genera were downloaded, and a phylogenetic tree was constructed using MEGA v11 with the neighbor-joining method and a bootstrap value of 1000 [9]. The cell morphology was observed using scanning electron microscopy (SEM) (MIRA3, TESCAN, Navi Innovation, Co., Ltd. (Changsha, China)). Prior to SEM observation, the samples were prepared following a previously published protocol [10]. Based on the above analysis, strain QBS3 was identified as a member of the genus *Acidithiobacillus* spp. and was designated *A. ferriphilus* QBS3.

### 2.3. Growth, Oxidation, and Bioleaching Study

QBS3 was inoculated into 100 mL liquid 9K medium (add 44.7 g/L FeSO_4_·7H_2_O as an energy substance). A separate 9K medium without bacteria was set up as a blank control. Culturing in a shaker (30 °C, 170 rpm), cultures were taken at 8 h intervals. The growth of QBS3 was indicated by the oxidation rate of Fe(II), which was detected using O-phenanthroline spectrophotometry [11]. The cell density was measured using the microscopic counting method, as the presence of massive jarosite hindered OD600 absorbance measurements.

In order to test the sulfur oxidation ability of QBS3, the strain was inoculated into 100 mL of liquid 9K medium with 1 g of sublimed sulfur powder as the energy source. The sulfur oxidation rate was detected by ICP-AES (SPECTRO BLUE, SPECTRO, Key Laboratory of Biohydrometallurgy of Ministry of Education (Changsha, China)), and the growth of QBS3 in the sulfur-containing medium was measured using OD600.

QBS3 was also used for sphalerite bioleaching. The sphalerite used in this study was detected by XRD (Rigaku, SmartLab, Navi Innovation, Co., Ltd. (Changsha, China)) and XRF (Zetium, PANalytical, Navi Innovation, Co., Ltd. (Changsha, China)), and the results showed that its elemental composition was 67.1% Zn, with trace amounts of Fe, and a purity higher than 99%. The sphalerite was ground to a particle size of 200–400 mesh through vibratory grinding prior to leaching. Bioleaching was carried out in 100 mL of liquid 9K medium with 8 g/L ferrous ion (as ferrous sulfate heptahydrate) added to improve bioleaching efficiency and allow for further investigation of the mechanism [12]. The bacterial inoculum volume was 10%. The acclimation process was initiated at 1% pulp density (*w*/*v*), and then increased to 4% to improve the bioleaching performance.

### 2.4. Whole Genome Sequencing, Assembly, and Annotation

*A. ferriphilus* QBS3 was inoculated into 9K medium and incubated at 30 °C for 3 days. The bacterial cells were collected by centrifugation according to the method described in Section 2.2. Jarosite must be completely removed to prevent its interference with subsequent DNA extraction. Collected bacterial cells were sent to Biomarker Technologies Co., Ltd. (Beijing, China) for whole genome sequencing. High-quality genomic DNA (OD260/280 = 1.8–2.0, total DNA ≥ 1 μg, concentration ≥ 40 ng/μL) was used for downstream library sequencing. Genome sequencing was performed using Oxford Nanopore Technologies (ONT) single-molecule real-time sequencing [13] combined with Illumina sequencing. Library construction was conducted using an SQK-LSK109 DNA-seq kit (Oxford Nanopore Technologies, Co., Ltd. (Oxford, UK)). Single-molecule library construction followed ONT protocols, including fragment selection, end-repair, nick-repair, and adapter ligation.

The raw third-generation sequencing data were quality-filtered. Low-quality reads, those with high N content and sequences shorter than threshold values after trimming, were removed to obtain 1,447,339,772 bp of high-quality clean reads. ONT data assembly was performed using Canu v1.5, and the assembly results were corrected using Racon v1.4.0 to assemble reads to generate contigs. Then, the assembled reads were judged into loops using Circlator software [14] to obtain the complete chromosome and plasmid genomes.

The coding sequences (CDSs) in the genome were predicted using Prodigal [15], and non-coding RNA was predicted using Infernal software [16]. The predicted CDSs were functionally annotated from GO (http://www.geneontology.org/, accessed on 5 January 2024), EggNOG (http://eggnog.embl.de/, accessed on 5 January 2024), and KEGG (http://www.genome.jp/kegg/, accessed on 5 January 2024).

### 2.5. Evolutionary Analyses and Functional Evaluation of Bioleaching-Related Genes

During bacterial evolution, exogenous function-coding genes can be acquired through transformation or transfection. These genes can be integrated into the bacterial genome and provide beneficial functions for bacteria. These exogenous gene fragments are called mobile genetic elements (MGEs). To better understand the MGEs in *A. ferriphilus* QBS3, in this study, we performed genomic island (GI) prediction using IslandViewer4 software, with IslandPath-DIMOB v0.2 as the selected prediction method [17]. Prophage regions were identified using Phispy V2.3 [18], while CRISPR-Cas elements were predicted using CRT V1.2 [19]. The ISFinder database was used to identify and classify insertion sequence (IS) elements in the genome [20]. The complete genome of *A. ferriphilus* QBS3 has been deposited in the NCBI database under accession numbers CP151686–CP151687.

### 2.6. Average Nucleotide Identity (ANI) and DDH Calculations

A comparative search of the NCBI database was performed, and the whole genome sequences of seven strains from *A. ferriphilus* sp. were downloaded as reference genomes. The average nucleotide identity based on MUMmer (ANIm) was calculated using the Kostas lab online tool (http://enve-omics.ce.gatech.edu/ani/index, accessed on 2 August 2024). In addition, digital DNA-DNA hybridization (dDDH) values between QBS3 and the reference strains were calculated using the TYGS strain genome server (https://tygs.dsmz.de/, accessed on 2 August 2024) [21]. DDH values are considered to be the most reliable criteria for delineating bacterial species relationships, alongside ANI and the percentage of conserved DNA. According to Goris et al., 70% DDH has been suggested as the cut-off point for species delimitation [22], corresponding to 95% ANI and 69% DNA conservation.

### 2.7. Pan-Genome Analyses of A. ferriphilus spp.

The total gene pool of a species can be much larger than that of a single strain. “Pan-genome” has been proposed to represent the full genetic repertoire of a bacterial species, including the core genome (genes present in all strains), the accessory genome (genes present in two or more strains), and unique genes specific to individual strains. Phylogenetic analysis was performed with the help of the IPGA v1.09 [23] on the *A. ferriphilus* genome, and both pan-genome and core-genome curves were generated.

The trend of contraction of the pan/core-genome can be determined based on the fitting models built into BPGA, the power-law model for the pan-genome (PS(n) = κnγ) and the exponential curve fitting model for the core genome (Fc(n) = κce(−n/τc) + Ω) [24], where κ, γ, κc, τc, and Ω are all free parameters. If γ < 0, it means that the size of the pan-genome gradually approaches a constant as the number of genomes increases. In other words, the pan-genome is closed; conversely, if 0 < γ < 1, the pan-genome is open.

## 3. Results

### 3.1. Isolation and Characterization of Bioleaching Bacteria

**Figure 1:**
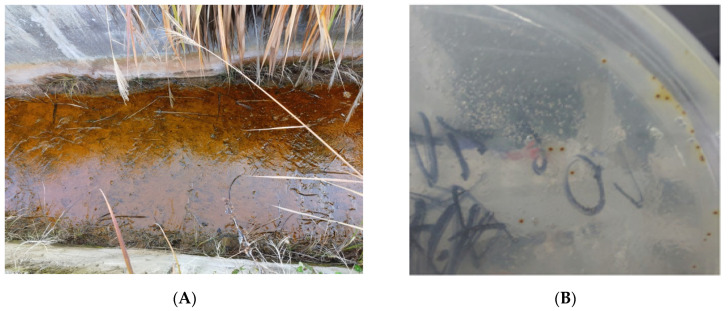

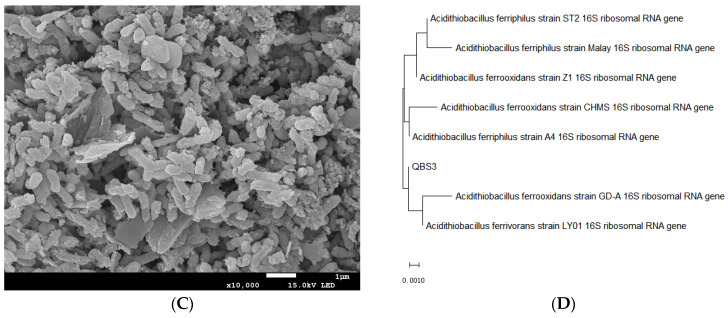
(**A**) Sampling site; (**B**) colony image; (**C**) bacterial cell SEM image; and (**D**) 16S rRNA gene phylogeny tree of QBS3 and seven closely related strains.

A bioleaching strain, QBS3, was isolated and purified from an acid mine drainage of the zinc–lead sulfide waste mine contaminated with multiple heavy metals from Qibaoshan Mine (Figure 1A). The colony morphology of QBS3 was small, dry, and red in color (Figure 1B), similar to the colony of *Acidithiobacillus* spp. [25]. The strain was found to be Gram-negative by Gram staining and to have a rod shape with a length of about 1.3 µm (Figure 1C), which was also similar to the bacteria morphology of *Acidithiobacillus* spp., which is commonly used in bioleaching [26]. BLAST analysis of QBS3’s 16S rDNA sequences showed a 99–100% similarity to *Acidithiobacillus* spp. The phylogenetic analysis of 16S rDNA further indicated that the strain is most closely related to *A*. *ferrooxidans*, *A. ferrivorans*, and *A. ferriphilus* (Figure 1D). Because the species in *Acidithiobacillus* spp. are closely related, identification based on the 16S rRNA gene alone is insufficient. After whole genome sequencing and dDDH analysis, strain QBS3 was ultimately identified as belonging to *A. ferriphilus*.

### 3.2. Physiological and Biochemical Characterization of QBS3

QBS3 can use ferrous ions and sulfur as energy sources, and has shown high ferrous and sulfur oxidation efficiency. It can completely oxidize 9 g/L ferrous ions within 24 h, reaching a plateau phase with a cell density of 9 × 10^7^ CFU/mL. When using sulfur as an energy source, the growth phase is longer, and the cell density at the plateau phase reaches 8 × 10^8^ CFU/mL.

**Figure 2:**
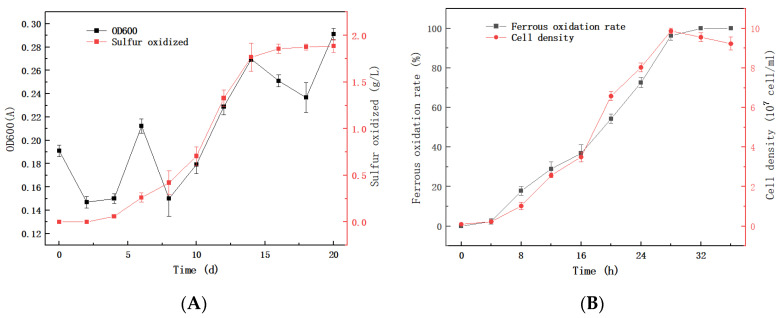

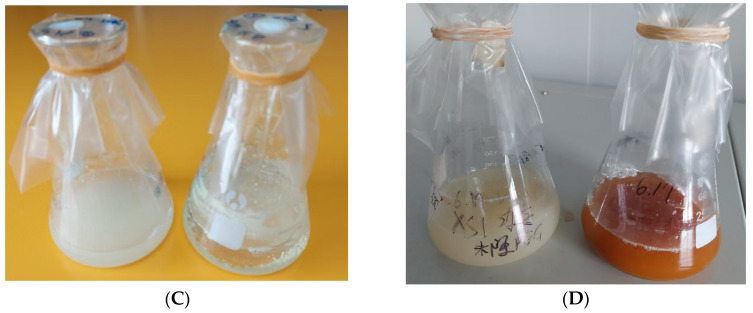
(**A**) Sulfur oxidation and growth curves of QBS3 in 10 g/L sulfur; (**B**) iron oxidation curve of QBS3 in 9 g/L Fe(II); (**C**) QBS3 cultured in 9K medium with the addition of 10 g/L sulfur; and (**D**) QBS3 cultured in 9K medium with the addition of 9 g/L Fe(II).

QBS3 has high ferrous oxidation activity due to its short lag phase and high oxidation rate in a logarithmic growth period. Its ferrous oxidation rate is 0.361 g/(L·h) in 24 h (Figure 2A), which is higher than the average reported for *Acidithiobacillus* strains [27], showing strong potential for bioleaching. Its sulfur oxidation activity is 0.206 g/(L·d) in 9 days (Figure 2B), comparable to that of a recently reported *A. ferrooxidans* strain [28]. Upon sulfur addition, the culture solution became turbid (Figure 2C). The addition of ferrous ions resulted in the formation of ferric ions and jarosite after incubation (Figure 2D).

### 3.3. The Sphalerite Bioleaching Effect of QBS3

**Figure 3:**
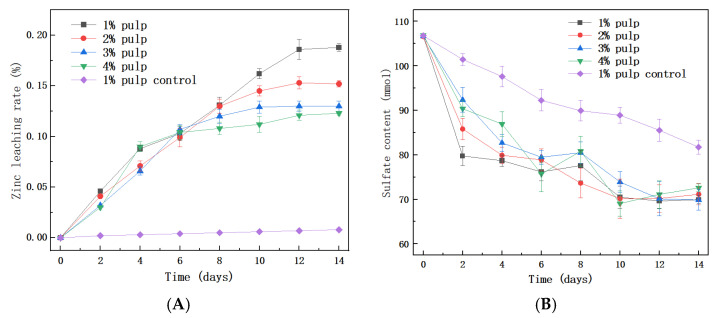

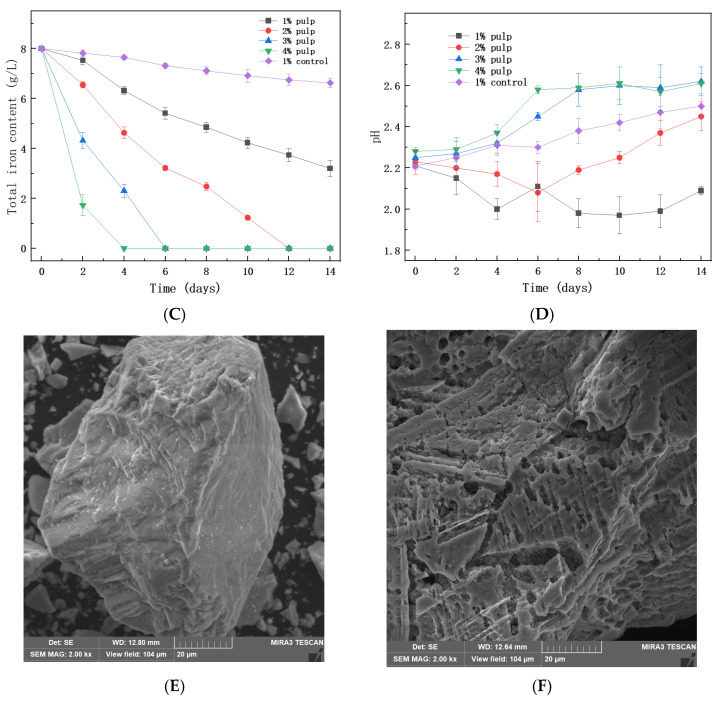
(**A**) Zinc leaching curve of 1% to 4% pulp sphalerite with QBS3; (**B**) sulfite content change curve; (**C**) total iron content change curve; (**D**) pH change curve; (**E**) SEM image of sphalerite before bioleaching; and (**F**) after bioleaching.

QBS3 was domesticated in 1% sphalerite pulp (9K medium) for two generations before being transferred to higher sphalerite pulp concentrations. In 1% to 4% pulp, QBS3’s zinc leaching process began immediately without a lag phase (Figure 3A). Simultaneously, the added ferrous ions were consumed rapidly, with no detectable ferrous ions remaining by day 2. Ferrous ions are readily utilized by *Acidithiobacillus* species, which helps eliminate the lag phase [29].

After 2 days of bioleaching, all ferrous ions were oxidized to ferric ions by QBS3. Since the pure sphalerite used in this study contains almost no iron, the ferric ions in the pulp did not increase. Instead, ferric ions precipitated in the form of jarosite (KFe_3_(SO_4_)_2_(OH)_6_). As the pulp density increased, the amount of sphalerite leached also increased; however, the sulfate content in the pulp remained similar (Figure 3B). This could explain the variation in total iron content observed at different pulp densities (Figure 3C). In higher pulp densities, ferric ions were depleted more quickly. Another reason could be pH changes—higher pulp densities consume acid faster, and a higher pH favors jarosite formation (Figure 3D). Scanning electron microscope (SEM) images of the residues revealed clear signs of surface corrosion (Figure 3E,F), indicating QBS3’s bioleaching capability.

It is worth noting that zinc leaching almost ceases when ferric ions are depleted. This may cause a lower zinc extract rate at higher pulp densities. After 14 days, zinc leaching efficiencies were 18.8% in 1% pulp density, 15.2% in 2% pulp, and 12–13% in 3% and 4% pulp. The bioleaching of iron-free pure sphalerite is challenging. A recent study reported a 10% zinc extract rate in 2% pulp after 23 days of bioleaching using mixed bioleaching strains with 10 g/L ferrous ion supplementation [30]. Another study reported a 3.38% zinc extract rate in 2% pulp after 16 days of bioleaching using *A. ferrooxidans* without ferrous ion supplementation [31]. This demonstrates that the supplementation of ferrous ions enhances the bioleaching of sphalerite. From a different perspective, although mixed strains generally show higher bioleaching efficiency [32], QBS3 achieved a similar zinc extraction rate within a shorter bioleaching period. This suggests that QBS3 possesses high bioleaching efficiency.

**Figure 4:**
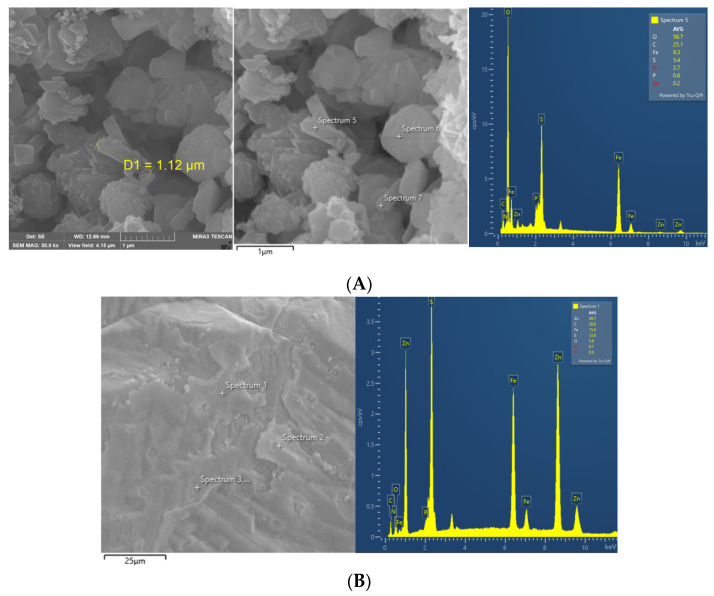
(**A**) SEM image and EDS analysis results of QBS3 cells attached to sphalerite; (**B**) biofilm on sphalerite.

Residue from 1% pulp density was used for SEM-EDS analysis, and QBS3 cells can be observed on the sphalerite particles (Figure 4A). A well-formed biofilm can also be observed (Figure 4B). The EDS analysis shows that the main composition on the particle surface is still sphalerite (ZnS), with no passivation layer. In previous zinc–sulfide ore bioleaching studies, jarosite was mainly described as having undergone passivation [33,34]. However, in this study, jarosite existed mainly in a loose form, and not wrapped around the ore particle. This finding is consistent with previous inferences that jarosite affects sphalerite bioleaching by exhausting ferric ions, rather than through a passivation effect.

### 3.4. Genomic Characteristics of QBS3

**Figure 5:**
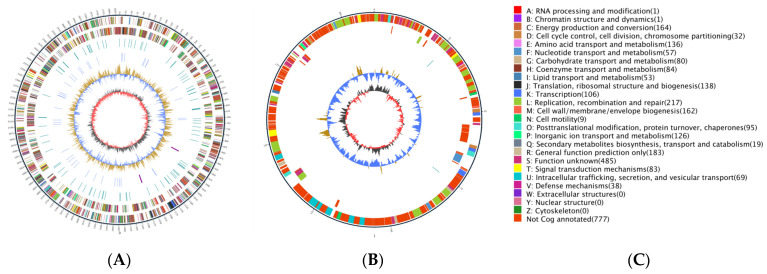
Circular genome map of *A. ferriphilus* QBS3. From outside to center: genes on the direct strand, genes on the complementary strand, tRNAs (blue), rRNAs (purple), GC content (yellow means higher than average, blue means lower), GC-skew. (**A**) Chromosome; (**B**) plasmid; (**C**) Gene annotation.

The genome size of *A. ferriphilus* QBS3 was 3,053,087 bp, including one circular chromosome and one circular plasmid. The sequence length of the circular chromosome was 2,842,766 bp, with a GC content of 57.10%, and the sequence length of the circular plasmid was 210,321 bp, with a GC content of 53.51%. A genome circle plot was created to illustrate the genome features (Figure 5). A total of 3115 coding sequences (CDSs), 6 rRNAs, and 50 tRNAs were predicted. The chromosome and plasmid were submitted to the GenBank database under accession numbers CP151686 and CP151687, respectively.

**Figure 6:**
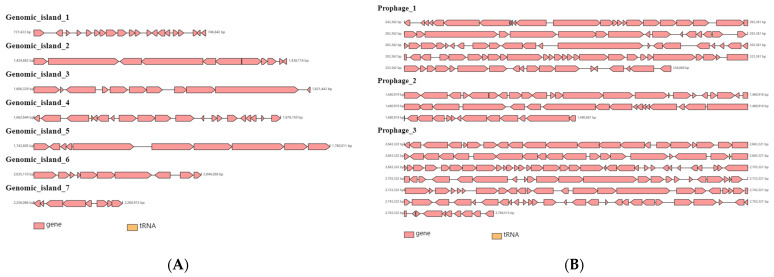
The genomic structures of genomic islands (**A**) and prophages (**B**) in the genome of *A. ferriphilus* QBS3.

Mobile gene elements (MGEs) are key drivers of genome evolution and play a critical role in horizontal gene transfer. Seven genomic islands, three prophages, and five CRISPRs were found in the QBS3 genome, as shown in Figure 6 and Table 1 and Table 2, indicating traces of horizontal gene transfer. Most of the MGEs of *A. ferripihlus* QBS3 are located on its chromosome.

**Figure 7:**
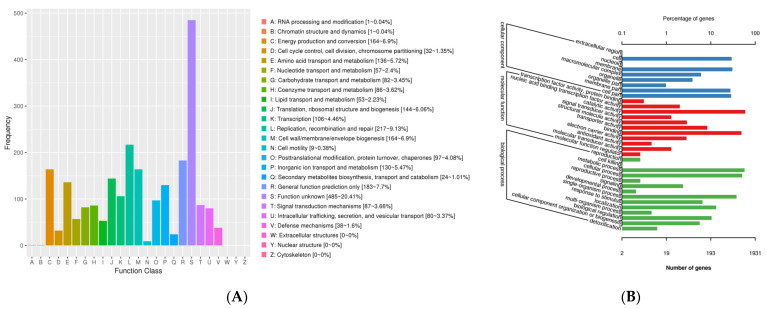
(**A**) eggNOG function classification; (**B**) GO function classification of the QBS3 genome.

The annotated results for QBS3 are as follows: 1931 genes annotated in the GO database, 1542 genes annotated in the KEGG database, and 2338 genes annotated in the eggNOG database. The eggNOG annotation (Figure 7) was divided into four categories and twenty-four functional classes. The proportion of genes related to replication, recombination, and repair (L), cell wall/membrane/envelope biogenesis (M), energy production and conversion (C), and inorganic ion transport and metabolism (P) was slightly higher than that of genes with other functions, accounting for 9.13%, 6.9%, 6.9%, and 5.47% of all annotated genes, respectively. This suggests that the QBS3 strain has a strong ability to resist extremely bioleaching environments.

### 3.5. Phylogenetic Analyses of QBS3

The 16S rRNA gene of QBS3 was compared in NCBI BLAST and found to be very similar to *A. ferriphilus* (99.93–100%), followed by *A. ferrooxidans* (99.15–99.93%) and *A. ferrivorans* (98.87–99.01%). Since a 98.7–99% similarity of the 16S rRNA gene is recommended as a threshold range for delineating new species, further comparison is needed to determine QBS3’s species.

**Figure 8:**
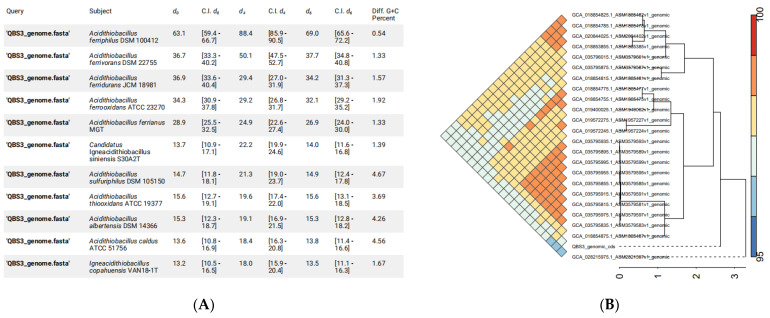
(**A**) DDH calculations between QBS3 and typical strains from the genus *Acidithiobacillus*; (**B**) ANI heatmap between QBS3 and other *A. ferriphilus* strains.

More accurate than 16s rRNA gene BLAST, researchers consider a 95–96% ANI value as the species boundary for prokaryotes, corresponding to 70% DDH [35]. The whole genome of QBS3 was compared with similar strains from *Acidithiobacillus* spp. As a result, 88.4% formula d4 dDDH and >97% ANI indicate that QBS3 is a new strain of *A. ferriphilus* species (Figure 8A). In the *A. ferriphilus* species, the ANI heatmap shows that strains QBS3 and YL25 are slightly more distantly related to other strains. For QBS3, SCUT-1 (GCA_019400025.1) and GF (GCA_035795915.1) are the most closely related strains (Figure 8B).

*A. ferriphilus* is closely related to *A. ferrooxidans*, which has been extensively studied and widely used in bioleaching. It is considered to have potential in the field of bioleaching and has not been previously used for leaching sphalerite.

**Figure 9:**
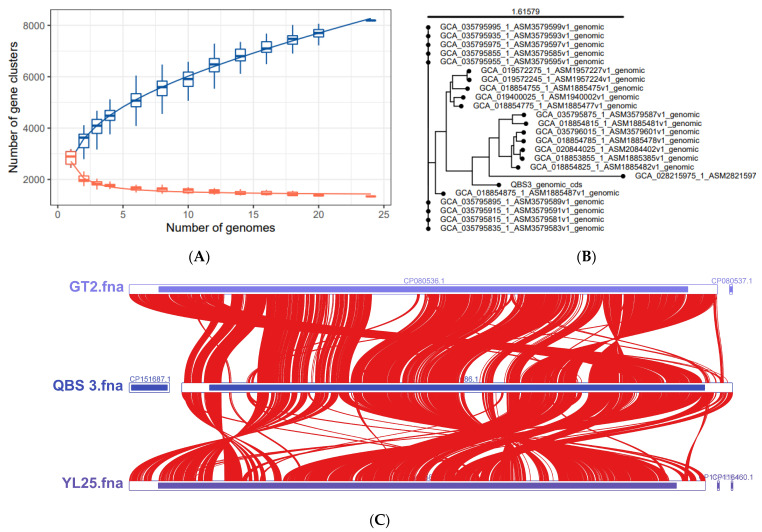
(**A**) Core gene rarefaction curve, Pan Gene (blue), Core gene(red); (**B**) whole genome phylogenetic tree of *A. ferriphilus*; and (**C**) synteny analysis result of strains QBS3, GT2, and YL25.

The pangenome analysis of QBS3 and 23 other reported *A. ferriphilus* strains was carried out using NMDC, yielding a total of 8194 pan-genomic gene counts. Of these, 1339 genes (16.34%) were core genes present in the genomes of all 24 strains. ZBY12 had the highest number of unique genes (380), while QBS3 had 163 unique genes. Because 0 < γ < 1, the pan-genome in this study is of the open type (Figure 9A). A phylogenetic tree was constructed based on the pangenome analysis (Figure 9B). QBS3 and YL25 were placed on a single branch, indicating that QBS3 and YL25 occupy separate evolutionary positions within *A. ferriphilus* strains. This may be due to the geographic locations where QBS3 and YL25 were isolated, which are distantly located from other reported strains. Synteny analysis was performed between genome-annotated strains QBS3, YL25, and GT2 (Figure 9C), showing a close relationship and traces of genomic structural variation.

### 3.6. Sphalerite Bioleaching-Related Genes of A. ferriphilus QBS3

*A. ferriphilus* QBS3 can use ferrous ions and various reductive inorganic sulfur compounds (RISCs) as electron acceptors to carry out aerobic oxidation, producing ferric ions and sulfuric acid during this process. Ferric ions and acid promote sphalerite bioleaching, and the related genes are key bioleaching function genes. In addition, the respiratory chain and carbon–nitrogen fixation functions also play an important role in QBS3 growth and environmental adaptation. The related genes are annotated on the NCBI and KEGG databases, which are listed below (Table 3). The plasmid sequence was also annotated; however, no genes associated with the bioleaching process were identified.

#### 3.6.1. Ferrous Oxidation-Related Genes

The ferrous oxidation pathway begins at the outer membrane, mainly occurs in the periplasmic, and ends in the inner membrane. Genes of related proteins located in these places are listed and contain Cyc2, Iro, RusA, RusB, CycA1, and Cyc1. Their respective functions are explained below.

The high molecular weight cytochrome c (Cyc2) is located in the outer membrane and has the ability to transfer electrons from ferrous ions to periplasmic proteins [36]. Iron oxidase (Iro) is also annotated in the QBS3 genome, located in the periplasmic, and can also transfer electrons from ferrous ions [37]. Rusticyanin has a high redox potential and acid stability, playing the role of an electron carrier [38]. Both types of rusticyanin (RusA and RusB) were annotated in the QBS3 genome. Cyc2 transfers electrons from ferrous ions to RusA [39], and Iro transfers electrons from ferrous ions to RusB.

In addition, periplasmic cytochrome c (CycA1) and cytochrome c (Cyc1) are also related to the ferrous oxidation electron transfer chain [39]. Electrons from RusA and RusB are transferred to CycA1 or Cyc1, and then transferred to the respiratory chain complex.

In summary, electrons from ferrous ions are taken by Cyc2 and Iro, and then transferred to RusA and RusB, respectively. RusA and RusB transfer electrons to CycA1 and Cyc1, and then transfer them to the respiratory chain complex.

#### 3.6.2. Sulfur Oxidation-Related Genes

Sulfur oxidation mainly occurs in the periplasm, helping QBS3 to maintain an acidic environment. Multiple types of enzymes related to the sulfur oxidation pathway were annotated in the QBS3 genome. Related genes are listed, including SQR, SoxB, SoxY, SoxZ, Trx, CycA2, TQO, and TetH. Their respective functions are explained below.

Sulfide:quinone reductase (SQR) was located in the inner membrane, and can oxidize sulfide to elemental sulfur [40] and transfer electrons to the quinone pool.

For elemental sulfur oxidation, QBS3 has an incomplete SOX system, including thiosulfate oxidation carrier (SoxYZ) and thiosulfohydrolase (SoxB). Rhodanese (Rho) and thioredoxin (Trx), located in the periplasmic space, can fill the gap, catalyzing the oxidation of elemental sulfur with SoxYZ and SoxB [40,41,42], generating sulfate and transferring electrons to the periplasmic cytochrome c (CycA2). Then, CycA2 transfers electrons to the respiratory chain complex.

Thiosulfate:quinone oxidoreductase (TQO) can oxidize thiosulfate to tetrathionate [43] and transfer electrons to the quinone pool. Then, tetrathionate hydrolase (TetH) hydrolyzes tetrathionate to sulfate and thiosulfate, meaning that thiosulfate can rejoin the cycle.

In summary, sulfide is oxidized to elemental sulfur by SQR. Elemental sulfur is oxidized to sulfate by an incomplete SOX system containing SoxYZ and SoxB; Trx and Rho can catalyze this process. Thiosulfate is oxidized by TQO.

#### 3.6.3. Respiratory Chain Complex-Related Genes

Electrons from iron sulfur oxidation are transferred to the quinone pool or respiratory chain complex and release energy. Cytochrome aa_3_ complex (cytochrome oxidase), cytochrome bo_3_ complex (ubiquinol oxidase), and cytochrome bc_1_ complex (ubiquinol–cytochrome c reductase) have been reported in *Acidithiobacillus* [44] and are also annotated in the QBS3 genome. The related genes are listed in Table 1 and contain two sets of genes encoding the aa_3_ complex, two sets of genes encoding the bo_3_ complex, and two sets of genes encoding the bc_1_ complex. In addition, two SDR genes and a HiPIP gene fall into this category. Their respective functions are explained below.

The aa_3_ complex can take electrons from Cyc1, CycA2 [45], and high-potential iron–sulfur protein (HiPIP) [46]. Electrons from ferrous ions and RISCs are terminally transferred to oxygen. The energy released in this way is used to transport protons out of the inner membrane, forming a proton gradient. The bo_3_ complex also transfers electrons to oxygen, but it takes electrons from the quinone pool instead of Cyc. Electrons are also transferred to oxygen, forming a proton gradient.

In QBS3, two different *pet* operons (*petI* and *petII*) encode two sets of the bc_1_ complex. For the *petI* operon, the bc_1_ complex takes part in ferrous oxidation, transferring electrons from CycA1 to the quinone pool. This step requires energy from the proton gradient because quinone has a lower redox potential, so this pathway is described as uphill electron transfer [47]. By comparison, the bc_1_ complex from the *petII* operon is transcribed for sulfur oxidation. It takes electrons from the quinone pool and transports protons out of the inner membrane, and then transfers electrons to HiPIP.

Two short-chain dehydrogenase/reductase (SDR) family genes were found in the QBS3 genome, annotated as YciK family oxidoreductase and NAD(P)-dependent oxidoreductase. They may take electrons from the quinone pool and transfer them to NADPH.

In summary, two sets of the aa_3_ complex, two sets of the bo_3_ complex, and two sets of the bc_1_ complex were annotated in the QBS3 genome. The respiratory chain complex takes electrons from iron–sulfur oxidation and makes them release energy.

#### 3.6.4. Carbon and Nitrogen Fixation-Related Genes

After the above pathways, energy from ferrous ions and RISCs is converted to a proton gradient. ATP synthase and the NDH complex (formerly the NAD(P)H dehydrogenase-like complex) utilize the energy derived from the proton gradient to drive the synthesis of ATP and NADPH [44], which are further consumed for carbon and nitrogen fixation. In the QBS3 genome, multiple sets of ribulose bisphosphate carboxylase/oxygenase (RubisCO) genes and one phosphoribulokinase (PrkB) are annotated and function as key enzymes in the Calvin cycle for carbon fixation [48]. Nitrogenase genes (Nif) are also annotated in the QBS3 genome, indicating that this strain has nitrogen fixation function.

## 4. Discussion

**Figure 10:**
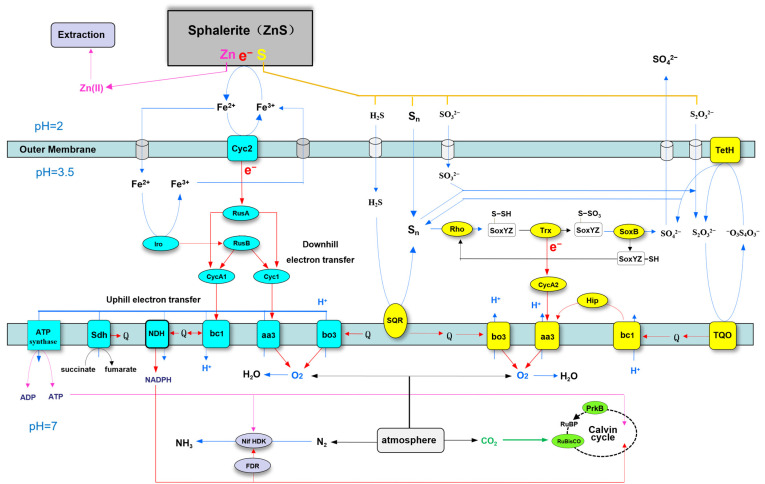
The putative mechanism model of sphalerite bioleaching by *A. ferriphilus* QBS3. The dissolution of sphalerite results from the oxidative attack of ferric ions (Fe^3+^) on the surface. In this interfacial redox reaction, ferric ions are reduced into ferrous ions (Fe^2+^) by the electrons from sphalerite, while the elements of Zn and S of sphalerite are gradually released into the bioleaching solution from the surface. The released soluble zinc ions (Zn^2+^) are extracted and thus achieve the industrial purpose of zinc metallurgy. The released sulfur may generate various reduced inorganic sulfur compounds (RISCs), such as hydrogen sulfide (H_2_S), polysulfur (S_n_), sulfite (SO_3_^2−^), thiosulfate (S_2_O_3_^2−^), etc., due to the complex reaction of sulfur chemistry. The role of *A. ferriphilus* in sphalerite bioleaching is to oxidize these ferrous ions and various RISCs. The oxidation of Fe^2+^ leads to the regeneration or recycling of the oxidant of Fe^3+^. The oxidation of RISCs results in the transformation of insoluble or inhibiting intermediates into the final highly soluble product of sulfate (SO_4_^2−^), thereby promoting the progress of sphalerite bioleaching. Meanwhile, the oxidation of spharelite by *A. ferriphilus* provides energy for the fixation of carbon dioxide (CO_2_) and nitrogen (N_2_) for the growth and reproduction of this chemoautotroph. The iron oxidation system of *A. ferriphilus* consists of downhill and uphill electron transfer pathways. The downhill pathway is from the oxidation of extracellular Fe^2+^ by Cyc2 or periplasmic Fe^2+^ by Iro, and then successively transfers electrons to rusticyanins (RusA and RusB), Cyc1, the aa_3_ complex, and O_2_. The uphill pathway pushed by the transmembrane gradient proton is bifurcated from rusticyanins (RusA and RusB), and then successively transfers electrons to CycA1, reverse bc_1_ complex, quinone pool, reverse NDH, and NADPH. The sulfur oxidation system of *A. ferriphilus* mainly contains SQR, Rho, SoxYZ, Trx, SoxB, TQO, TerH, etc. The various extracellular RISCs enter the periplasmic space via the outer membrane or pore channels. SQR catalyzes H_2_S to produce sulfane sulfur (S_n_) and puts an electron into the quinone pool. SoxYZ binds the sulfur with the help of Rho and is then oxidized by Trx to produce SoxYZ-thiosulfate adduct and bring an electron to the aa_3_ complex via CycA2. SoxB splits this adduct to generate the final product of sulfate. Sulfur and sulfite can non-enzymatically and reversibly be placed into thiosulfate. TQR catalyzes thiosulfate to produce tetrathionate (O_3_^2−^SSSSO_3_^2−^) and puts electrons into the quinone pool. TetH hydrolyzes tetrathionate to produce thiosulfate, sulfur, and sulfate. The forward bc_1_ complex extracts electrons from the quinone pool and then transfers them to the aa_3_ complex via Hip to reduce oxygen into water. The bo_3_ complex directly extracts electrons from the quinone pool to reduce oxygen. Under the actions of various respiratory components, the transmembrane proton gradient is formed and thus drives ATP synthetase to produce ATP. The carbon fixation is realized by the Calvin cycle with the energies of ATP and NADPH. The nitrogen fixation is performed by the nitrogenase system (NifHDK and FDR) under the energy of ATP and NADPH.

In this study, a high-iron–sulfur oxidation efficiency bioleaching strain, *A. ferriphilus* QBS3, was isolated from zinc-rich tailings and used for pure sphalerite bioleaching. The zinc extraction rate was 18.8% after 14 days. Zinc bioleaching mainly occurred before ferric ions were exhausted. The whole genome of QBS3 was sequenced, annotated, and analyzed. Based on the bioleaching results and bioleaching-related genes annotated in the QBS3 genome, a putative mechanism model of sphalerite bioleaching by *A. ferriphilus* QBS3 was proposed (Figure 10).

The iron (ferrous) oxidation pathway begins at the outer membrane. Ferrous ions are oxidized to ferric ions by Cyc2 and Iro. Ferric ions can oxidize sphalerite and be reduced to ferrous ions and rejoin the cycle. This is consistent with the results of the sphalerite bioleaching experiment; zinc leaching slows significantly after ferric ions are exhausted. For QBS3, the electrons from the ferrous ions are transferred by two pathways: Cyc2→RusA [39] and Iro→RusB. The Iro→RusB pathway does not exist in *A. ferrooxidans* and could be an important characteristic of *A. ferriphilus* [49]. Then, electrons from RusA and RusB are transferred to CytochromeA1 (CycA1) and Cytochrome1 (Cyc1), which determine the direction of the following electron transfer (uphill or downhill).

Electrons transferred to CycA1 enter uphill electron transfer and are taken by the bc_1_ complex and transferred to the quinone pool. Cyc1 transfers electrons to the aa_3_ complex. Electrons transferred this way enter downhill electron transfer and are finally transferred to oxygen [50]. The energy released from downhill electron transfer is used to transport protons out of the inner membrane, forming a proton gradient. ATP synthase uses the proton motive force from the gradient to synthesize ATP for the Calvin cycle.

Low-valent sulfur from ZnS oxidized by ferric ions is transported to the periplasmic space. Then, sulfide is oxidized to elemental sulfur by SQR [51], transferring electrons to the quinone pool. Elemental sulfur is transported to the sulfhydryl group of the SoxYZ complex by Rho, forming a disulfide bond [52]. The disulfide on SoxYZ is oxidized by Trx to a sulfo group, transferring electrons to CycA2 [53]. The sulfo group on SoxYZ is converted to sulfate by SoxB [54], and then SoxYZ rejoins the cycle.

Elemental sulfur reacts with sulfite in the periplasm and forms thiosulfate. TQO in the inner membrane oxidizes thiosulfate to tetrathionate, transferring electrons to the quinone pool [55]. TetH in the outer membrane decomposes tetrathionate into elemental sulfur, sulfite, and sulfate [56]. Elemental sulfur and sulfite can rejoin the sulfur oxidation process [57].

Electrons from the quinone pool are transferred to the bo_3_ complex, and then transferred to oxygen, releasing energy and transporting protons out of the inner membrane. Alternatively, they can be transferred to the bc_1_ complex, and then transferred to the aa_3_ complex by high-potential iron–sulfur protein (HiPIP) [46]. Electrons from CycA2 are transferred to complex aa_3_ as well.

In general, the iron–sulfur oxidation mechanism of *A. ferriphilus* QBS3 takes place in accordance with previous reports of the genus *Acidithiobacillus*. QBS3 has a reductive inorganic sulfur compound (RISC) oxidation pathway similar to that of *A. ferrooxidans* ATCC23270, including TQO and TetH. But in elemental sulfur oxidation, without the important enzyme sulfur dioxygenase (SDO) in ATCC23270, QBS3 may have a different pathway. Rho and Trx complement the incomplete Sox system and establish the elemental sulfur oxidation cycle. In iron oxidation, an Iro-related pathway was denied in ATCC23270 because most strains of *A. ferrooxidans* do not have the Iro gene [58]. However, both Iro and HiPIP were found in the QBS3 genome, revealing that this pathway (Iro→Rus) may also exist in QBS3. This may explain the strong iron oxidation ability (0.361 g/(L·h)) of QBS3.

The present study has certain limitations in the experimental verification of genes. The metabolic pathway needs further research on gene expression in QBS3. In future studies, experiments based on transcriptomics could be helpful.

This study isolated a high-iron–sulfur oxidation efficiency bioleaching strain, *A. ferriphilus* QBS3, and used QBS3 in sphalerite bioleaching. Whole genome sequencing and analysis revealed the iron–sulfur-oxidation pathways of QBS3, contributing to the zinc sulfide ore bioleaching industry and iron–sulfur-oxidation mechanism studies.

## 5. Conclusions

In this study, a high-bioleaching-efficiency strain (*A. ferriphilus* QBS3) was screened. QBS3 was used for pure sphalerite bioleaching, which is challenging and has been seldom studied. Zinc was effectively leached with the addition of ferrous iron in the early phase. However, when ferric ions were exhausted by forming jarosite, the leaching was hindered. The whole genome of *A. ferriphilus* QBS3 was sequenced and analyzed through pangenome analysis, showing traces of horizontal gene transfer. Bioleaching-related genes were annotated. Based on bioleaching results and gene annotation, a hypothetical sphalerite bioleaching pathway map for QBS3 was drawn. While many mechanisms are similar to *A. ferrooxidans*, some unique features were found in QBS3. Without SDO, QBS3 may oxidize elemental sulfur through Rho, Trx, and an incomplete SOX system. Iro and HiPIP are also important in both iron and sulfur oxidation. A limitation of this study is the lack of experimental validation of the genes. Future transcriptomic studies could address this gap and further elucidate the metabolic mechanisms of QBS3. A hypothetical sphalerite bioleaching model is proposed, providing a theoretical basis for bioleaching.

## Figures and Tables

**Table 1:** The statistics of genomic islands and prophages in the genome of *A. ferriphilus* QBS3.

Name	Location	Start	End	Length
Genomic_island_1	Chromosome	737,433	746,842	9410
Genomic_island_2	Chromosome	1,424,882	1,438,714	13,833
Genomic_island_3	Chromosome	1,606,329	1,621,443	15,115
Genomic_island_4	Chromosome	1,662,644	1,676,150	13,507
Genomic_island_5	Chromosome	1,743,805	1,760,011	16,207
Genomic_island_6	Chromosome	2,035,110	2,044,268	9159
Genomic_island_7	Chromosome	2,256,066	2,260,915	4850
Prophage_1	Chromosome	243,362	338,889	95,528
Prophage_2	Chromosome	1,440,919	1,490,881	49,963
Prophage_3	Chromosome	2,643,322	2,768,515	125,194

**Table 2:** The statistics of CRISPRs in the genome of *A. ferriphilus* QBS3.

Name	Location	Start	End	Repeat Number	Average Repeat Length (bp)	Spacer Number	Average Spacer Length (bp)
CRISPR.1	Chromosome	2,434,984	2,435,070	2	33	1	21
CRISPR.2	Chromosome	2,604,343	2,604,427	2	19	1	47
CRISPR.3	Chromosome	2,662,550	2,662,633	2	24	1	36
CRISPR.4	Plasmid	51,317	51,420	2	28	1	48
CRISPR.5	Plasmid	169,386	169,782	6	36	5	36

**Table 3:** The NCBI and KEGG database predicts genes associated with sphalerite bioleaching in *A. ferriphilus* QBS3.

Name	Gene Locus	Length (AA)	Annotation
**Ferrous oxidation-related genes**			
Iro	AAE485_08910	90	Iron oxidase, high potential iron–sulfur protein (HiPIP) family, secreted
RusB	AAE485_10410	189	B-type rusticyanin, secreted, secreted
RusA	AAE485_10875	187	A-type rusticyanin, secreted, secreted
Cyc1	AAE485_10910	229	c-type cytochrome, secreted, secreted
Cyc2	AAE485_10915	487	Outer membrane cytochrome c, secreted
CycA1	AAE485_00725	260	c-type cytochrome, secreted
**Sulfur oxidation-related genes**			
SQR	AAE485_13635	394	Sulfide:quinone reductase (SQR), FAD-dependent oxidoreductase, secreted
DoxA	AAE485_01905	172	Thiosulfate:quinone oxidoreductase (TQO) subunit, DoxA domain-containing protein
DoxD	AAE485_08680	333	Thiosulfate:quinone oxidoreductase (TQO) subunit, DoxD
CycA2	AAE485_14115	237	c-type cytochrome, secreted
TetH	AAE485_03710	503	Tetrathionate hydrolase (TetH), PQQ-binding-like beta-propeller repeat protein, The Outer Membrane Protein Insertion Porin (Bam Complex) (OmpIP) Family, secreted
SoxB	AAE485_06770	575	Thiosulfohydrolase SoxB, SignalP, secreted
SoxZ	AAE485_06780	110	Thiosulfate oxidation carrier complex protein SoxZ, secreted
SoxY	AAE485_06785	170	Thiosulfate oxidation carrier protein SoxY, secreted
Rho	AAE485_07140	141	Rhodanese-like domain-containing protein, The Sulfate Permease (SulP) Family, secreted
Trx	AAE485_03190	194	Thioredoxin_2 domain, secreted
Trx	AAE485_07965	206	Disulfide isomerase, Thioredoxin_2 domain, secreted
Trx	AAE485_02165	394	Pyridine nucleotide–disulfide oxidoreductase, secreted
Trx	AAE485_09980	406	Pyridine nucleotide–disulfide oxidoreductase, secreted
**Respiratory chain complex-related genes**			
CoxD	AAE485_10885	64	Cytochrome C oxidase subunit IV, aa_3_ complex
CoxC	AAE485_10890	184	cbb_3_-type cytochrome c oxidase subunit III, aa_3_ complex
CoxA	AAE485_10895	627	cbb_3_-type cytochrome c oxidase subunit I, aa_3_ complex
CoxB	AAE485_10900	254	Cytochrome C oxidase subunit II, aa_3_ complex
CoxD	AAE485_09335	184	Cytochrome C oxidase subunit IV, aa_3_ complex
CoxC	AAE485_09330	627	cbb_3_-type cytochrome c oxidase subunit III, aa_3_ complex
CoxA	AAE485_09325	254	cbb_3_-type cytochrome c oxidase subunit I, aa_3_ complex
CoxB	AAE485_10900	254	Cytochrome C oxidase subunit II, aa_3_ complex
CyoD	AAE485_12980	128	Cytochrome O ubiquinol oxidase, bo_3_ complex
CyoC	AAE485_12985	212	Cytochrome c oxidase subunit 3, bo_3_ complex
CyoB	AAE485_12990	715	cbb_3_-type cytochrome c oxidase subunit I, bo_3_ complex
CyoA	AAE485_05135	310	Ubiquinol oxidase subunit II, bo_3_ complex
CyoD	AAE485_13040	118	Cytochrome O ubiquinol oxidase, bo_3_ complex
CyoC	AAE485_13045	211	Cytochrome c oxidase subunit 3, bo_3_ complex
CyoB	AAE485_13050	677	cbb_3_-type cytochrome c oxidase subunit I, bo_3_ complex
CyoA	AAE485_12995	321	Ubiquinol oxidase subunit II, bo_3_ complex
Sdr1	AAE485_00730	262	SDR family NAD(P)-dependent oxidoreductase
PetA1	AAE485_00735	206	Ubiquinol–cytochrome c reductase iron–sulfur subunit, bc_1_ complex
PetB1	AAE485_00740	402	Cytochrome b N-terminal domain-containing protein subunit, bc_1_ complex
PetC1	AAE485_00745	245	Cytochrome c1 subunit, bc_1_ complex
Sdr2	AAE485_03975	272	SDR family NAD(P)-dependent oxidoreductase
PetA2	AAE485_03980	206	Ubiquinol–cytochrome c reductase iron–sulfur subunit, bc_1_ complex
PetB2	AAE485_03985	404	Cytochrome b N-terminal domain-containing protein subunit, bc_1_ complex
PetC2	AAE485_03990	232	Cytochrome c1 subunit, bc_1_ complex
Hip	AAE485_03995	104	High potential iron–sulfur protein (HiPIP) family, secreted
**Carbon fixation-related genes**			
rbcS	AAE485_12075	118	Ribulose bisphosphate carboxylase (RubisCO) small subunit
rbcL	AAE485_04495	473	Form I ribulose bisphosphate carboxylase (RubisCO) large subunit
rbcL	AAE485_09375	473	Form I ribulose bisphosphate carboxylase (RubisCO) large subunit
rbcS	AAE485_04490	110	Ribulose bisphosphate carboxylase (RubisCO) small subunit
rbcL	AAE485_12300	459	Ribulose bisphosphate carboxylase (RubisCO) acetyl-CoA pathway
rbcS	AAE485_09380	110	Ribulose bisphosphate carboxylase (RubisCO) small subunit
rbcL	AAE485_12080	473	Form I ribulose bisphosphate carboxylase (RubisCO) large subunit
prkB	AAE485_06610	290	Phosphoribulokinase
**Nitrogen fixation-related genes**			
nifK	AAE485_09730	519	Nitrogenase molybdenum–iron protein subunit beta
nifD	AAE485_09735	491	Nitrogenase molybdenum–iron protein alpha chain
nifH	AAE485_09740	296	Nitrogenase iron protein

## Data Availability

The complete genome of *A. ferriphilus* QBS3 was deposited in the NCBI database under accession numbers CP151686–CP151687.

## Abbreviations

The following abbreviations are used in this manuscript:

XRD	X-ray Diffraction
XRF	X-ray Fluorescence
ICP	Inductively Coupled Plasma
CRISPR	Clustered Regularly Interspaced Short Palindromic Repeats
